# A High-Fat Diet Induces Low-Grade Cochlear Inflammation in CD-1 Mice

**DOI:** 10.3390/ijms23095179

**Published:** 2022-05-06

**Authors:** Jeffrey Chan, Ravi Telang, Dagmara Kociszewska, Peter R. Thorne, Srdjan M. Vlajkovic

**Affiliations:** Department of Physiology and The Eisdell Moore Centre, Faculty of Medical and Health Sciences, The University of Auckland, Private Bag 92019, Auckland 1142, New Zealand; jeffrey.chan@auckland.ac.nz (J.C.); r.telang@auckland.ac.nz (R.T.); d.kociszewska@auckland.ac.nz (D.K.); pr.thorne@auckland.ac.nz (P.R.T.)

**Keywords:** gastrointestinal tract, inner ear, high-fat diet, cochlear inflammation, gut dysbiosis, hearing loss

## Abstract

There is growing evidence for a relationship between gut dysbiosis and hearing loss. Inflammatory bowel disease, diet-induced obesity (DIO), and type 2 diabetes have all been linked to hearing loss. Here, we investigated the effect of a chronic high-fat diet (HFD) on the development of inner ear inflammation using a rodent model. Three-week-old CD-1 (Swiss) mice were fed an HFD or a control diet for ten weeks. After ten weeks, mouse cochleae were harvested, and markers of cochlear inflammation were assessed at the protein level using immunohistochemistry and at the gene expression level using quantitative real-time RT-PCR. We identified increased immunoexpression of pro-inflammatory biomarkers in animals on an HFD, including intracellular adhesion molecule 1 (ICAM1), interleukin 6 receptor α (IL6Rα), and toll-like-receptor 2 (TLR2). In addition, increased numbers of ionized calcium-binding adapter molecule 1 (Iba1) positive macrophages were found in the cochlear lateral wall in mice on an HFD. In contrast, gene expression levels of inflammatory markers were not affected by an HFD**.** The recruitment of macrophages to the cochlea and increased immunoexpression of inflammatory markers in mice fed an HFD provide direct evidence for the association between HFD and cochlear inflammation.

## 1. Introduction

Epidemiological studies suggest that a high body mass index (BMI) in the obesity range, and to a lesser extent, in the overweight range, is positively associated with hearing loss [1,2,3,4,5,6,7]. Sensorineural hearing loss (SNHL) has also been reported as an extra-intestinal manifestation of inflammatory bowel disease (IBD) [8,9,10]. The pathogenesis of IBD includes a genetic predisposition to a defective mucosal barrier resulting in innate immune responses to the luminal contents [11]. Increased gut permeability caused by IBD permits the entry of bacterial or viral antigens from the gut microbiome into the systemic circulation; this can induce systemic inflammation [11]. Circulating inflammatory mediators (cytokines and chemokines) may compromise the permeability of the blood–labyrinth barrier (BLB) by stripping off its protective glycocalyx [12]. As the inner ear responds to immune challenges, systemic inflammation can result in the infiltration of inflammatory cells and mediators from the systemic circulation and deposition of inflammatory complexes in the inner ear. IBD has been associated with gut dysbiosis and significantly increased levels of bacterial plasma membrane components such as lipopolysaccharide (LPS) [13,14,15]. In the murine cochlea, systemic injection of low-dose systemic LPS increases the number of infiltrative monocytes into the cochlea [16]. 

The host’s diet is one of the most potent modulators of the gut microbiome. The presence of particular nutrients in a host’s diet may directly confer a competitive advantage to specific phyla of bacteria, which may lead to specific dietary changes in the composition of the gut microbiome [17]. The host’s diet may also indirectly induce changes in the gut microbiome by affecting host immunity and metabolism. These imbalances in the composition of the gut microbiome are called gut dysbiosis. Chronic HFD is often associated with gut dysbiosis, a compromised mucosal firewall, and chronic systemic inflammation [18,19]. 

Approximately 90% of the gut microbiota derives from Bacteroidetes and Firmicutes bacteria [20]. Chronic HFD is a well-established model of gut dysbiosis in mammals by disturbing the ratio between Firmicutes and Bacteroidetes [15,21,22]. Interestingly, gut dysbiosis can occur in the absence of obesity [23]. Multiple processes facilitate gut dysbiosis induced by an HFD, including increased oligo-and monosaccharide components associated with an HFD and decreased gut pH [22,24]. These changes to nutritional availability and the acidity in the gut foster an environment that allows Firmicutes to gain a survival advantage over Bacteroidetes. This disbalance also reduces overall bacterial diversity in the gut microbiome [22].

The gut barrier function is dependent on the relationship between commensal gut bacteria, the gut mucosal firewall, and the host immune system. It is well established that an HFD can alter the gut microbiota and thus induce gut barrier dysfunction [25,26,27]. Gut barrier dysfunction results from dynamic interactions between the gut microbiota and the innate immune system of the gut, along with metabolic changes associated with the shift in gut microbiota populations [28].

Cochlear innate immunity has an important role in mediating bacterial inflammation as part of an integrated defence system against exogenous stressors. Inflammation is initiated by pathogen recognition receptors that recognize and bind to pathogen-associated molecular patterns and damage-associated patterns [29]. The activation of these receptors leads to the upregulation of inflammatory cytokine expression, followed by the recruitment of resident and circulating leukocytes to the site of inflammation. These leukocytes phagocytose cellular debris and foreign antigens and induce the apoptosis of damaged cellular structures [15]. The BLB limits the extravasation of infiltrating leukocytes and foreign antigens from the circulation to reduce cochlear damage from inflammation [30]. However, recent evidence suggests that increased permeability of the BLB also occurs during cochlear inflammation, which impacts the ability of the BLB to limit the entry of immune cells and inflammatory or infectious agents into the cochlea [30,31,32]. This can potentially exacerbate cochlear damage to cause SNHL.

Herewith, we postulate that gut dysbiosis induced by an HFD may cause low-grade chronic inflammation in the cochlea. To test this hypothesis, we investigated the effect of a chronic HFD on the expression of inflammatory mediators in the cochlea using a mouse model. We used qRT-PCR to detect gene expression of inflammatory markers in the cochlea such as Toll-like receptors 2 and 4 (TLR2, TLR4), intracellular adhesion molecule 1 (ICAM1), interleukin 6 (IL-6), tumor necrosis factor-alpha (TNF-α), transforming growth factor-beta (TGF-β) and immunohistochemistry to detect protein expression of ICAM1, TLR2, interleukin 6 receptor α (IL6Rα), and the occurrence of ionized calcium-binding adapter molecule 1 (Iba1) positive migratory macrophages [33]. ICAM1, TLRs, and inflammatory cytokines are all associated with the nuclear factor kappa-light-chain-enhancer of the activated B cells (NF-κB) inflammatory signalling pathway [34,35]. TLR2 is activated in pathogen-mediated cochlear inflammation [36], whilst TLR4 expression can also be induced by sterile cochlear inflammation after acoustic injury or exposure to ototoxic drugs such as aminoglycosides or cisplatin [37,38]. Both receptors are upregulated during intestinal inflammation [39]. ICAM1 is an adhesion molecule that interacts with receptors on the surface of the immune cells to enable their extravasation into the cochlea [34,35]. IL-6 and TNF-α are markers of acute inflammation, while TGF-β is associated with the resolution phase of inflammation [40]. 

## 2. Results

### 2.1. Body Weight

Bodyweights (BW) were measured in all mice before and after completing the 10-week long diet regime. The baseline BW was measured at three weeks of age and the post-diet BW at 13 weeks. As expected, the mice on HFD showed significantly higher weight gain than mice on the control diet (CD) (9.6 g vs. 2.8 g; Table 1). Male and female mice in the HFD group showed similar weight gain (males: 29.1% ± 3.4; females: 28.4% ± 2.8).

### 2.2. ICAM1 Immunolabelling in the Cochlea of CD-1 Mice on a Chronic HFD 

ICAM1 is an adhesion molecule critical for the extravasation of leukocytes from the peripheral circulation to the site of inflammation. Previous studies have identified the lateral wall (spiral ligament and stria vascularis) as the region with the most consistent ICAM1 immunoexpression in the cochlea [34,41,42]. 

**Figure 1:**
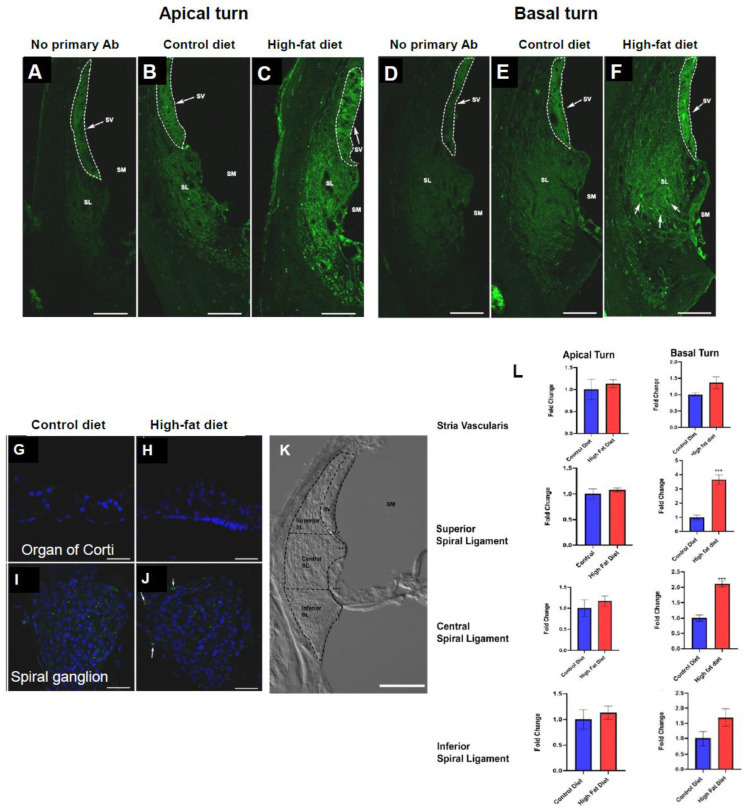
ICAM1 immunofluorescence in the mouse cochlea. (**A**,**D**). A low-level autofluorescence was observed in the stria vascularis in control cochlear cryosections where the primary antibody was replaced with the normal mouse IgG conjugated with Alexa 488. (**B**,**E**) In mice on a control diet (CD), weak ICAM1 immunostaining was observed in the spiral ligament (SL) and stria vascularis (SV) facing scala media (SM). (**C**,**F**) ICAM1 immunoexpression in the cochlear lateral wall (SV and SL) increased in intensity in mice on a high-fat diet (HFD). (**G**–**I**) ICAM1 immunofluorescence was not observed in the organ of Corti and the spiral ganglion of mice on CD. (**J**) Mice on HFD showed localised ICAM1 fluorescence in the spiral ganglion mostly around blood vessels (arrows). (**K**) Demarcations of the lateral wall used for semi-quantitative analysis of ICAM1. (**L**) Relative fold differences in ICAM1 immunostaining intensity in the lateral wall of the apical and basal cochlear turns. Data presented as mean ± SEM (*n* = 5 per diet group). *** *p* < 0.001, *t*-test. Scale bars, 50 μm. Blue, DAPI; Green, ICAM1 immunofluorescence.

ICAM1 immunofluorescence in the lateral wall was observed consistently in both turns of the mouse cochlea (apical and basal) and both diet groups (Figure 1B,C,E,F). Negative control (no primary Ab), where the ICAM1-Alexa 488 antibody was replaced with normal mouse IgG directly conjugated with Alexa 488, was included in each experiment. In control cryosections, we observed weak autofluorescence in the stria vascularis and the spiral ligament (Figure 1A,D). ICAM1 immunofluorescence was absent from the sensory and supporting cells of the organ of Corti (Figure 1G,H) and spiral ganglion neurons in CD mice (Figure 1I). In mice on HFD, localised ICAM1 immunoexpression was observed at the periphery of the spiral ganglion, likely associated with blood vessels (Figure 1J; arrows).

The relative ICAM1 immunostaining intensity was assessed semi-quantitatively using ImageJ in both turns of the mouse cochlea. Mid-modiolar cryosections were used for assessment with a total of five cochleae from different animals per diet group. The spiral ligament was divided into three segments (superior, central, and inferior), while the stria vascularis was analysed in its entirety. The boundaries of the spiral ligament regions were made based on anatomical landmarks, as shown in Figure 1K. Pixel intensity in the stria vascularis and spiral ligament were normalised to the background signal in the scala media. The relative intensity of ICAM-1 immunostaining was similar between the diet groups in the apical turn, but different in the basal turn (Figure 1L). The relative intensity of ICAM1 immunolabelling in the superior and central spiral ligament of the basal turn was significantly (3.6 and 2.1 fold increase, respectively, *p* = 0.0001 and 0.00004, *t*-test) higher in the HFD group than the CD group. Immunofluorescence intensity was similar in the stria vascularis and the inferior region of the spiral ligament in both turns (Figure 1L).

### 2.3. IL6Rα Immunolabelling in the Cochlea of CD-1 Mice on a Chronic HFD 

IL-6 is promptly and transiently produced in response to infections and tissue injuries, contributing to host defense by stimulating acute-phase responses and immune reactions [43]. Changes in IL-6 expression are often associated with changes in IL-6 receptor expression [43]. Our study demonstrated differential IL-6Rα immunolabelling in the cochleae of mice on different diet regimens. IL-6Rα immunolabelling was similar in both turns of the cochlea in each diet group. 

**Figure 2:**
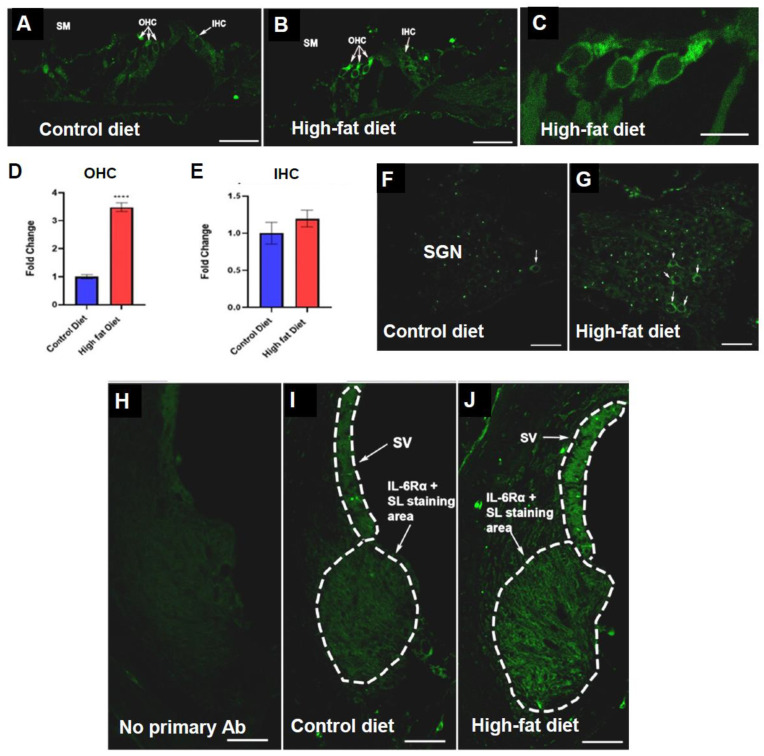
IL-6Rα immunofluorescence in the mouse cochlea. (**A**,**B**) immunostaining in the organ of Corti (OoC). (**A**) Weak IL-6Rα immunofluorescence was detected in the sensory and supporting cells of the OoC in mice on the control diet (CD). (**B**,**C**) In mice on HFD, IL-6Rα immunofluorescence was intense in the outer hair cells (OHC). (**D**) Semi-quantitative analysis of immunofluorescence intensity in OHC showed a 3.5 fold increase in mice on a high-fat diet (HFD), whilst the immunofluorescence intensity in the inner hair cells (IHC) was similar in CD and HFD mice (**E**). Data presented as mean ± SEM (*n* = 5 per diet group). **** *p* < 0.0001, *t*-test. (**F**,**G**) In the spiral ganglion, more neurons expressed IL-6Rα in mice on HFD (arrows). (**I**,**J**) IL-6Rα immunofluorescence was observed in lateral wall tissues (stria vascularis and the spiral ligament) in both diet groups, whilst the background signal was very low in control cochlear cryosections (**H**). The area and intensity of immunofluorescence were slightly increased in the lateral wall of the mice on HFD, but this was not statistically significant. Scale bars, 30 μm (**A**,**B**,**F**,**G**); 10 μm (**C**); 50 μm (**H**–**J**).

The organ of Corti of mice on HFD showed enhanced IL-6Rα immunolabelling compared to CD mice, particularly in the outer hair cells (OHC) (Figure 2A,B). IL-6Rα immunofluorescence in the OHC was predominantly cytoplasmic, in the perinuclear and supranuclear regions (Figure 2C). Semi-quantitative analysis of IL-6Rα immunofluorescence was performed by measuring relative pixel intensity (ImageJ) in the OHC. The three rows of OHCs within the organ of Corti were averaged to generate a mean OHC grey value. These values were calculated for mid-modiolar sections of 10 animals (*n* = 5 per diet group). In mice on an HFD, the relative staining intensity in the OHC increased 3.5 fold compared to the CD group (*p* = 0.0000005, *t*-test; Figure 2D). In contrast, the intensity of IL-6R immunolabelling in the inner hair cells (IHCs) did not differ significantly between the two diet groups (Figure 2E). 

IL-6Rα immunofluorescence was also observed in the spiral ganglion, housing the primary auditory neurons of the cochlea. More neurons appeared to express IL-6Rα in mice on an HFD (Figure 2F,G); however, the average immunofluorescence intensity was not statistically different between the spiral ganglions in HFD and CD mice (data not shown). The control cryosections incubated with the normal IgG-Alexa 488 conjugate instead of the primary antibody showed a very low background level in the lateral wall of the cochlea (Figure 2H). In contrast, IL-6Rα immunofluorescence was observed in the stria vascularis and spiral ligament in both diet groups (Figure 2I,J). The area and intensity of immunofluorescence were slightly increased in mice on HFD, but this was not statistically significant (data not shown). 

### 2.4. TLR2 Immunolabelling in the Cochlea of CD-1 Mice on a Chronic HFD

Toll-like receptor 2 (TLR2) is an integral receptor in the bacterial inflammation pathway, responsible for initiating the pro-inflammatory cascade [36]. 

**Figure 3:**
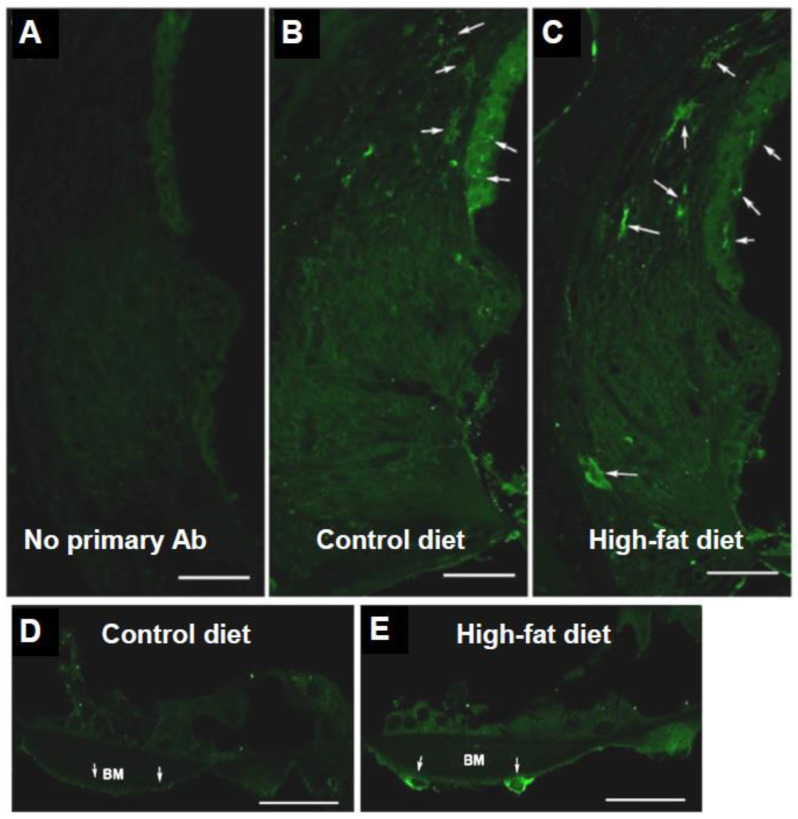
TLR2 immunofluorescence in the mouse cochlea. (**A**) control section stained with the normal IgG-Alexa 488 conjugate; (**B**,**C**) TLR2 immunofluorescence in the lateral wall of the cochlea in mice on a high-fat or control diet; (**E**) TLR2 immunofluorescence was observed in the mesothelial cells (arrows) under the basilar membrane (BM) in mice on a high-fat diet (HFD), but not in mice on a control diet (**D**). Scale bars, 50 μm (**A**–**C**), 35 μm (**D**,**E**).

TLR2 immunolabelling was primarily observed in the lateral wall of the cochlea in both diet groups (Figure 3B,C), with a similar TLR2 immunofluorescence pattern in the apical and basal turns. There was a minimal background signal in the lateral wall when the primary antibody was replaced by the normal IgG-Alexa488 conjugate (Figure 3A). In CD mice, TLR2 immunofluorescence was mainly concentrated in the upper spiral ligament region, adjacent to the stria vascularis (arrows; Figure 3B). In HFD mice, TLR2 was also identified further laterally and inferiorly in the spiral ligament (arrows; Figure 3C), but the overall differences in immunofluorescence intensity, however, were not significant (data not shown). TLR2 immunofluorescence was also observed in the mesothelial cells lining the scala tympani in mice on an HFD (arrows, Figure 3E), but not in CD mice (Figure 3D). No TLR2 immunostaining was observed in the spiral ganglion in either diet group (data not shown). 

### 2.5. Iba1-Positive Cells in the Cochlea of CD-1 Mice on a Chronic HFD 

Iba1 is a well-established macrophage marker expressed upon cell activation. Iba1 labels both peripherally recruited and resident cochlear macrophages [44]. 

**Figure 4:**
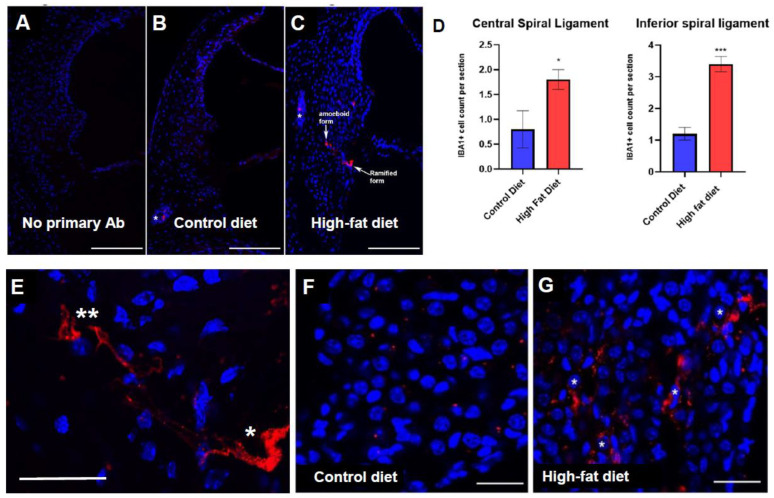
Iba1+ macrophages in the mouse cochlea. (**B**,**C**) Iba+ cells were observed in both diet groups but not in control cryosections stained with a normal IgG-Alexa 594 conjugate (**A**). In mice on a control diet (**B**), rare Iba1+ cells were located at the spiral ligament/otic capsule border (asterisk). In mice on a high-fat diet (**C**), Iba1+ cells were found mainly within the inferior spiral ligament (arrows). (**D**) The number of Iba1+ cells in HFD mice was higher than in CD mice in the central and inferior regions of the spiral ligament. Data presented as mean ± SEM (*n* = 5 per diet group). * *p* < 0.05; *** *p* < 0.001; *t*-test. (**E**) Iba1+ macrophages displayed both ramified (*) and amoeboid (**) morphologies. (**F**,**G**) Iba1+ cells were also found in the spiral ganglion, predominantly in the HFD group (**G**). Scale bars, 100 μm (**A**–**C**), 30 μm (**E**–**G**).

This study reveals the presence of Iba1+ macrophages in the cochleae of both diet groups, although their distribution was different (Figure 4B,C). In CD mice, rare Iba1+ cells were located at the spiral ligament/otic capsule border (asterisk; Figure 4B). In mice on an HFD, Iba1+ cells were found mainly within the inferior spiral ligament (arrows; Figure 4C). These macrophages display both ramified (homeostatic) and amoeboid (activated) morphologies (Figure 4E). The number of Iba1+ cells were counted in each region of interest (ROI) and averaged across the middle optical slices from the z-stack to obtain an Iba1+ cell count per section for a given ROI. An Iba1+ cell was defined by the co-expression of Iba1 and DAPI (nuclear staining). The number of Iba1+ cells in HFD mice was higher than in CD mice in both the central and inferior regions of the spiral ligament (Figure 4D). Iba1+ cells were also found in the spiral ganglion (Figure 4F,G), predominantly in the HFD group. These Iba+ cells cluster around the neurons with processes extending to the cellular surroundings (Figure 4G). 

### 2.6. Gene Expression of Inflammatory Biomarkers in the Cochlea of CD-1 Mice on a Chronic HFD 

A quantitative real-time RT-PCR study was performed to quantify gene expression levels of pro- and anti-inflammatory markers in mice on an HFD. This study evaluated differences in transcript levels of key pro-inflammatory biomarkers in the cochlea (TLR2, TLR4, TNF-α, IL-6, and ICAM1), and the expression of TGF-β was assessed as an anti-inflammatory biomarker. For this study, 12 mouse cochleae were used in total (*n* = 6 per diet group), each obtained from a different animal. The differences in transcript levels of the target genes between the two dietary groups were quantified relative to the housekeeping gene, β-actin, using the 2^-^^ΔΔCt^ method [45]. The 2^-^^ΔΔCt^ method expresses the differences in transcript levels as a fold change in gene expression relative to the CD group. Statistical differences between the two dietary groups were assessed using the Student’s *t*-test. 

**Figure 5:**
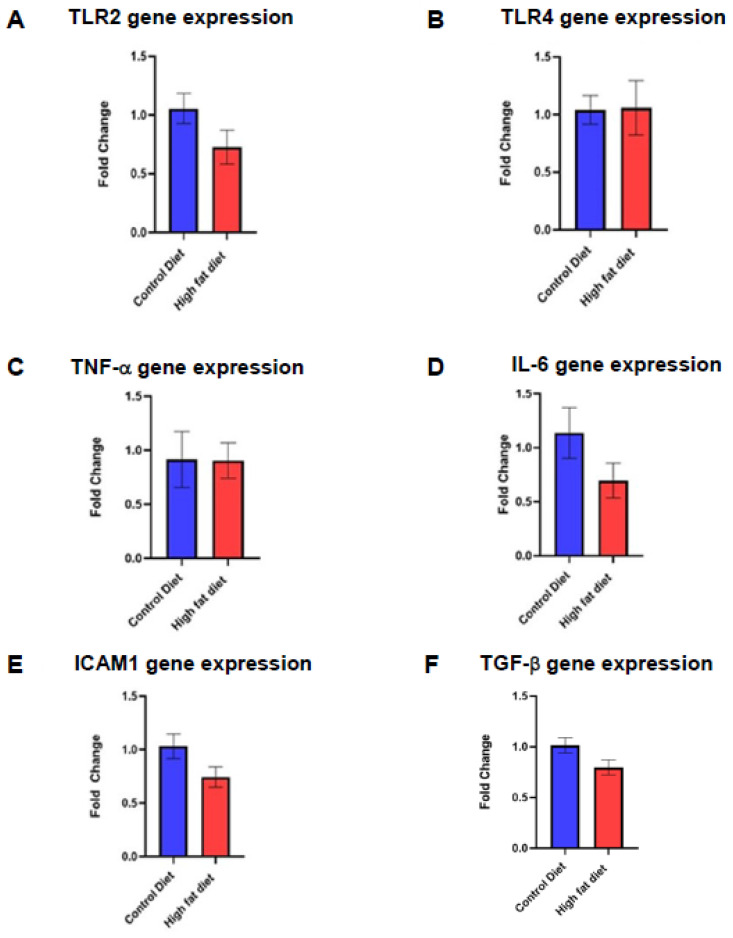
Quantitative gene expression studies in the mouse cochlea (qRT-PCR). (**A**–**F**) Gene expression levels of toll-like receptors (TLR2, TLR4), inflammatory cytokines (TNF-α, IL-6), adhesion molecule ICAM1, and anti-inflammatory mediator TGF-β were compared in mice on a high-fat diet (HFD) and control diet (CD). There were no significant differences in the gene expression of these mediators between HFD and CD mice. Data presented as mean ± SEM (*n* = 6 per diet group).

The relative gene expression levels for TLR2, TLR4, IL-6, TNF-α, ICAM1, and TGF-β were similar in HFD and CD groups (Figure 5). No amplification was detected in control -RT reactions and when the reaction template (cDNA) was omitted (data not shown).

## 3. Discussion

The study used immunohistochemistry and quantitative real-time RT-PCR to assess changes in cochlear inflammatory biomarkers at the protein and gene expression level. 

At the protein level, pro-inflammatory biomarkers ICAM1 and IL-6Rα were upregulated in the mouse cochlea in response to a chronic HFD. TLR2 immunofluorescence was observed in the mesothelial cells lining the scala tympani in mice on an HFD. The infiltration of Iba1+ macrophages was observed in the spiral ganglion and the spiral ligament in mice on a chronic HFD. At the transcript level, there were no significant differences between animals on an HFD and CD in the gene expression of pro-inflammatory and anti-inflammatory biomarkers. This may indicate that changes in inflammatory biomarkers are regulated at the protein level rather than the gene expression level. Alternatively, changes in gene expression levels induced by HFD were transitory and therefore not captured at the study’s endpoint. 

### 3.1. ICAM1 Protein Expression in the Cochlea of CD-1 Mice on a Chronic HFD 

ICAM1 is a vascular adhesion molecule expressed in the endothelial cells of arterioles and venules [41]. The function of ICAM1 is to facilitate leukocyte transmigration from the periphery into the tissue during inflammation. In this study, ICAM1 immunostaining was identified in the lateral wall of mice on a high-fat and control diet, but the immunofluorescence was enhanced in the spiral ligament of mice on HFD. In the HFD group, ICAM1 immunostaining increased in the superior and central spiral ligament in the basal turn of the cochlea but not in the apical turn. 

It is well established that the basal turn of the cochlea is anatomically and physiologically different from the apical turn. The basal turn is more sensitive to oxidative stress [46], and the cochlear base-to-apex metabolic gradients were observed in various forms of cochlear pathology (e.g., age-related hearing loss, noise- and drug-induced hearing loss). High-frequency hearing loss is the most common pattern of sensorineural hearing loss, providing strong evidence for the increased vulnerability of the cochlear basal turn to metabolic stress and injury. Increased ICAM-1 immunoexpression in the basal turn suggests that this region could respond more to inflammatory challenges than the apical turn. However, immunoexpression of other inflammatory biomarkers, IL-6Rα and TLR2, were similar in both cochlear turns, suggesting that turn-related differences in response to an HFD were relatively subtle.

This expression pattern is similar to enhanced ICAM1 immunoexpression during cochlear inflammation resulting from noise exposure, labyrinthitis, immune response, and physical trauma [34,35,42,47,48]. In the present study, the inferior portion of the spiral ligament in the basal turn of the cochlea was mostly devoid of ICAM1 immunolabelling, whereas, in noise-exposed C57BL/6 mice, it was the region with the strongest ICAM1 immunoexpression [34]. This difference can be explained by the significant degeneration of type IV fibrocytes in the inferior spiral ligament of the basal turn that occurs in CD-1 mice [4,49], which a chronic HFD may further exacerbate. Degeneration of type IV fibrocytes starts at approximately three weeks of age in CD-1 mice and is progressive [50]. These mice show accelerated presbyacusis with hearing loss commencing at around four weeks [49]. A possible cause is the spiral ligament damage in the basal turn that precedes hair cell loss, while other studies report that spiral ganglion degeneration occurs first [50]. In our study, type IV fibrocyte degeneration was evident in the basal cochlear turn in both control and HFD mice at 13 weeks (endpoint of the study). As a result of cellular degeneration in the inferior spiral ligament, ICAM1 immunoexpression in this region was not observed in our study. Early degeneration of fibrocytes in this region also raises the possibility that macrophages were recruited to the spiral ligament to remove cell debris. However, increased occurrence of Iba1+ macrophages in the inferior and central spiral ligament of mice on HFD relative to control mice suggests that HFD enhanced macrophage recruitment to the cochlea.

Accelerated presbyacusis in CD-1 mice thus represents a limitation for these studies. However, the enhanced inflammation in CD-1 mice on HFD compared to mice on the control diet indicates a separate cochlear effect of the HFD and systemic inflammation in these relatively young mice (three months old). A longitudinal study using the CD-1 mouse model could help understand the interactions between age-related changes and chronic systemic inflammation. Future studies should investigate the effect of HFD in other mouse strains that show late-onset age-related hearing loss (e.g., CBA mice) and extend to other small and large animal models to investigate the consistency of these findings across mammalian species. 

**Figure 6:**
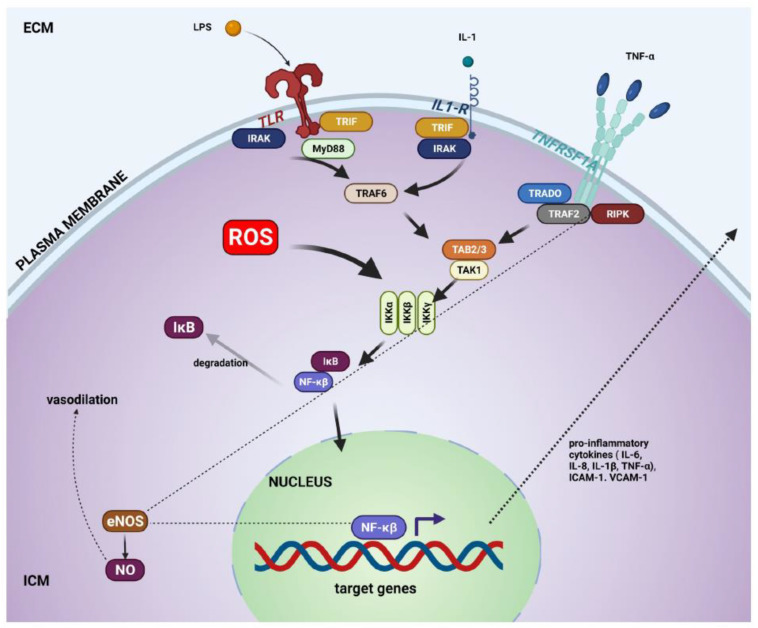
Canonical NF-κB signalling pathway. NF-κB pathway regulates inflammatory responses, apoptosis, and cell growth. Extracellular pro-inflammatory cytokines and bacterial metabolites (LPS) can activate the pathway through appropriate receptors (such as toll-like receptors, IL-1R, and TNFRSF1A) and by an accumulation of intracellular reactive oxygen species (ROS). These stress signals regulate the downstream IKK kinase complex. Activated IKKβ subunit phosphorylates IkB, which then is ubiquitinated and marked for degradation by proteases. This discharges NF-κB from the IkB-containing complex and uncovers the nuclear localisation signal (NLS). Then, NF-κB complex translocases to the nucleus, where it triggers the transcription of target genes that will produce pro-inflammatory cytokines and intracellular adhesion molecules [34,51,52]. Intracellular adhesion molecule-1 (ICAM-1) and vascular cell adhesion molecule (VCAM1) facilitate the infiltration of leukocytes from the bloodstream into injured tissue during inflammation [53]. These adhesion molecules hold the leukocytes in close proximity to the endothelial cells to allow receptor/ligand interactions and facilitate leukocyte diapedesis between endothelial cells [35]. In the cochlea, ICAM-1 is expressed in the basal cells of the stria vascularis, type II and IV fibrocytes of the spiral ligament and endothelial cells of the spiral modiolar vein [35]. As a result, ICAM-1 has a critical role in regulating the cochlear immune response by facilitating the extravasation of peripheral leukocytes into the cochlea during inflammation: ECM, extracellular matrix; eNOS, endothelial nitric oxide synthase; ICM, intracellular matrix; IRAK, Interleukin-1 receptor-associated kinase; NO, nitric oxide; TRAF, TNF receptor-associated factor; TRADD (TNF-R1-associated death domain protein); TRIF, TIR-domain-containing adapter-inducing interferon-β.

The significant upregulation of ICAM1 in mice on an HFD is consistent with the increased leukocyte transmigration from the peripheral circulation into the spiral ligament. ICAM1 expression, induced in cochlear fibrocytes by pro-inflammatory cytokines such as TNF-α, is presumably mediated by NFκB activity [34,51]. The activation of NF-κB was demonstrated previously in the spiral ligament and spiral ganglion of CD-1 mice with HFD-induced obesity [4]. Furthermore, signal transducer and activator of transcription proteins (STAT) signalling induced by the activation of IL-6R receptor can also induce ICAM1 expression [52]. NF-κB and STAT signalling pathways may thus work synergistically to upregulate ICAM1 in the spiral ligament in response to a chronic HFD. The canonical NF-κB pathway is illustrated in Figure 6.

### 3.2. Protein Expression of IL-6Rα in the Cochlea of CD-1 Mice on a Chronic HFD 

The IL-6 receptor (IL-6R) consists of the IL-6Rα ligand-binding chain and GP130 signal transducing chain [54]. IL-6 binds to the IL-6R α-subunit and, subsequently, the signal transduction domain, GP130, leading to the activation of the intracellular signalling pathway [43]. 

In our study, CD-1 mice on HFD displayed enhanced IL-6Rα immunoexpression in the cochlea compared to mice on the control diet. IL-6Rα immunoexpression was particularly increased in the OHCs of the organ of Corti. In addition, qualitative differences in IL-6Rα immunoexpression were also observed in the spiral ligament and spiral ganglion. The observed pattern of cochlear IL-6R expression in mice on HFD resembles the pattern observed in noise-induced cochlear inflammation, with IL-6R upregulation in the spiral ligament, sensory hair cells, supporting cells and spiral ganglion neurons following acoustic overstimulation [55]. These findings indicate that the cochlea increases its sensitivity to IL-6 in response to a chronic HFD. IL-6R-mediated intracellular signalling pathways activate inflammatory genes essential to macrophage recruitment, including monocyte attracting chemokine proteins and cell adhesion molecules like ICAM1 [56,57]. The upregulation of IL-6Rα may thus indicate a need to increase the expression of downstream inflammatory genes facilitating the recruitment of effector immune cells into the cochlea in response to a chronic HFD. 

Furthermore, IL-6R upregulation in the OHCs suggests that an HFD contributes to cochlear inflammation via activation of IL-6R in sensory hair cells. The organ of Corti often shows the signs of sensory cell injury during cochlear inflammation, where the OHCs are more prone to insult and display signs of degeneration before the IHCs [58]. 

### 3.3. Protein Expression of TLR2 in Response to a Chronic HFD

TLR2 expression has a key role in initiating the pro-inflammatory response to bacterial labyrinthitis [36,59]. The activation of TLR2 in the spiral ligament fibrocytes plays a crucial role in the upregulation of chemokines such as the monocyte chemotractant proteins, which guide the migration of infiltrating macrophages into the cochlea [36]. The immunoexpression of TLR2 in mesothelial cells of the basilar membrane in our study is consistent with TLR4 immunoexpression in these cells in response to acoustic injury and associated sterile inflammation of the cochlea [37]. The spiral ligament and the mesothelial cells of the basilar membrane are both sites within the cochlea that are in direct contact with macrophages. The spiral ligament is perfused by a vast capillary network that contains circulating leukocytes and is a common site of peripheral immune cell recruitment during both sterile and bacterial cochlear inflammation [59,60,61,62]. The basilar membrane mesothelial cells are also in direct contact with the macrophages in the scala tympani [63,64]. The upregulation of TLR2 in this area may indicate a signal to recruit nearby macrophages in response to the increased presence of TLR-activating ligands such as lipopolysaccharide (LPS) and lipoteichoic acid resulting from a chronic HFD [28].

### 3.4. Iba1+ Cell Expression in the Cochlea of CD-1 Mice on a Chronic HFD

Iba1 is a calcium-binding protein associated with the membrane ruffling process during phagocytosis in monocytes and macrophages [65]. Previous studies have identified Iba1 as a reliable macrophage marker in the cochlea [44,66]. 

We observed infiltration of Iba1+ cells in the spiral ligament and spiral ganglion in mice on HFD. The Iba1+ cell counts in the spiral ligament were higher in the HFD group than in the CD group. The location and appearance of these cells in the cochlea were similar to that observed in previous studies of cochlear inflammation induced by acoustic over-stimulation, infection and physical trauma [44,57,66,67]. Cochlear inflammation in those studies was characterised by the increased number of Iba1+ macrophages, particularly in the inferior region of the spiral ligament and the spiral ganglion, similar to our study. The number of Iba1+ cells in HFD group was lower compared to the recruitment of Iba1+ cells in response to surgical stress [44], suggesting that a chronic HFD induces low-grade cochlear inflammation. 

Overall, the increased number of Iba1+ cells in the spiral ligament suggests that a chronic HFD increases macrophage recruitment to the cochlea. 

### 3.5. Gene Expression of Inflammatory Biomarkers in the Cochlea of Mice on a Chronic HFD

Whilst the immunofluorescence study demonstrated an upregulation of inflammatory mediators in mice on an HFD, this was not linked to gene expression changes. It is well established that changes in mRNA expression do not always correlate at the protein level given the delay between transcription and translation, post-transcriptional modifications to mRNA, and differences in the half-life of proteins relative to mRNA [68]. In our study, the changes in transcript levels of inflammatory mediators likely occurred prior to changes at the protein level and returned to normal by the time when the cochleae were harvested. Another caveat is that the real-time qRT-PCR was performed on whole cochlear lysates, which means that subtle regional changes in gene expression could have been missed. However, as the immunoexpression of inflammatory markers was observed in all regions of the cochlea (lateral wall, organ of Corti, spiral ganglion), it would require cochlear decapsulation and manual tissue dissection, which are technically challenging in small rodents and typically results in loss of tissues.

Similar studies of HFD-induced inflammation in the mouse hypothalamus [69,70] have shown that, in response to an acute HFD, pro-inflammatory cytokines are upregulated initially due to increased activation of toll-like receptors and then suppressed to prevent extensive damage from the inflammatory process. This neuroprotective response is eventually exhausted, allowing chronic inflammation to develop [69,70]. The cochlea may also develop a dynamic inflammatory response to an HFD, with the outcome depending on the duration of the diet. There is increasing evidence that the loss of sensorineural tissues in the cochlea is exacerbated by inflammation [33]. Infiltrating immune cells and their pro-inflammatory cytokines, as well as reactive oxygen species (ROS), lead to a bystander tissue injury and may cause irreversible damage to delicate cochlear structures [33]. Cells of the innate immune system are the first to respond to inflammation. Macrophages and neutrophils aim to kill damaged cells by releasing ROS, and to remove dying and dead cells by phagocytosis. The next stage of acute inflammation is the infiltration of T lymphocytes, which represent the adaptive immune response. T cells may recognize self-antigens as a result of the cellular debris arising from damage and inflammation [33]. The resolution of inflammation is an active process that occurs after the acute phase of inflammation. During the resolution phase of inflammation, which promotes tissue repair, pro-resolution mediators such as lipoxins, resolvins, protectins, and maresins are released [40]. Pro-resolution mediators combine both anti-inflammatory and pro-resolving activities. They inhibit the release of pro-inflammatory cytokines, limit infiltration of neutrophils, enhance macrophage uptake, and stimulate clearance of apoptotic neutrophils and microbial particles. Further studies are therefore required to demonstrate dynamic changes in the cochlear inflammatory response to an HFD relative to the duration of diet and animal species.

In summary, HFD can cause gut dysbiosis, hyperpermeability of the intestinal barrier, and infiltration of pathogens and microbial metabolites into the systemic circulation [28]. Systemic inflammation can disrupt the integrity of the blood–labyrinth barrier (BLB), facilitating the infiltration of pathogens and pro-inflammatory cytokines into the inner ear [28]. Cochlear inflammation affects the ability of the BLB to limit the entry of immune cells and inflammatory agents into the cochlea, which can lead to chronic low-grade cochlear inflammation [30,31]. There is considerable evidence that acute or chronic inflammatory processes in the inner ear can exacerbate or induce hearing loss. In our recent review [71], we presented evidence from clinical studies that sensorineural hearing loss is one of the common extraintestinal manifestations of the inflammatory bowel disorders. In addition, viral or bacterial labyrinthitis (inflammation of the inner ear) is a common cause of temporary or permanent hearing loss. Many studies support the idea that the inflammatory process is also intimately associated with drug-, noise- and age-related hearing loss (40). This evidence points to the important link between unresolved cochlear inflammation and hearing loss. However, the link between HFD and hearing loss remains to be firmly established in future pre-clinical and clinical studies.

## 4. Materials and Methods

### 4.1. Animals

In this study, 3-week old CD-1 (Swiss) mice of both sexes were obtained from the animal facility at the University of Auckland. The animals were housed in cages containing nesting material and corn-cob bedding. Between two and five animals were housed per cage, with males and females housed separately. All experimental procedures were approved by the University of Auckland Animal Ethics Committee.

### 4.2. High-Fat Diet

For ten weeks, a group of 3-week old mice (*n* = 15) were fed a high-fat diet (HFD; Research Diets, New Brunswick, NJ, D12492I) consisting of 60% kilocalories of fat (Supplementary Figure S1). The control mice (*n* = 15) were placed on a regular chow diet (CD) that contained 10% kilocalories of fat (Research Diets, New Brunswick, NJ, USA, D12450J; Supplementary Figure S2), also for ten weeks. The animals were randomly assigned to each diet group. The food hopper was quarter filled and renewed every three days to maintain the freshness of the food. Baseline weight measurements were obtained at three weeks of age, and the final weight was measured at 13 weeks. 

### 4.3. Tissue Preparation

After the 10-week diet, all animals were euthanized using anesthetic overdose (pentobarbitone, 90 mg/kg intraperitoneally). The animals were perfused through the heart with the flush solution (0.9% NaCl containing 10% NaNO_2_) followed by whole-body perfusion with 4% paraformaldehyde (PFA) in 0.1 M phosphate buffer (PB, pH 7.4). The cochleae were dissected from the temporal bone, fixed in 4% PFA overnight at 4 °C, and decalcified using 5% EDTA in 0.1 M PB for nine days at 4 °C. The cochleae were then cryoprotected in 30% sucrose in 0.1 PB overnight and embedded in the Tissue-Tek^®^ Optimal Cutting Temperature (OCT) compound Sakura^®^ (Torreance, CA, USA). The cochleae were immediately snap-frozen and stored at −80 °C. 

### 4.4. Immunohistochemistry

Confocal immunofluorescence was used to characterise immunoexpression and distribution of inflammatory mediators in the mouse cochlea. Immunoexpression of toll-like receptor 2 (TLR2), interleukin 6 receptor (IL-6Rα), intracellular adhesion molecule 1 (ICAM1), and macrophage marker Iba1 was investigated in the lateral wall of the cochlea, organ of Corti, and spiral ganglion. The IL-6Rα, ICAM1, and Iba1 primary monoclonal antibodies were directly conjugated to either Alexa Fluor 488 or 594 fluorescent dyes, whilst the TLR2 was unconjugated. Each antibody was titrated to obtain an optimal concentration (Table 2). 

### 4.5. Immunohistochemistry for IL-6Rα, ICAM1, and Iba1

The cochleae (*n* = 15 per diet group) were cryosectioned at 20 μm using the Leica CM3050 S Cryostat (Leica Microsystems, Wetzlar, Germany) and placed in 48-well plates containing 0.1 M phosphate-buffered saline (PBS). Mid-modiolar cochlear cryosections (on average, four per cochlea) were obtained from 5 different animals from each diet group. Cochlear cryosections were washed with 0.1M PBS (3 × 10 min), permeabilized with 1% Triton X-100 and blocked with UltraCruz Blocking reagent (Santa Cruz Biotechnologies, Dallas, TX, USA) for 1 h at room temperature. The cochlear cryosections were incubated overnight at 4 °C with the conjugated antibody in UltraCruz Blocking reagent containing 0.1% Triton X-100. The next day, immunolabelled cryosections were washed in PBS (2 × 10 min and the final 40-min wash) and mounted on a glass slide using Fluoroshield^TM^ with DAPI mounting medium (Sigma-Aldrich, St. Louis, MO, USA). In control reactions, the primary antibody was replaced with the normal mouse IgG directly conjugated with Alexa 488 (Santa Cruz, sc-516606) or Alexa 594 (Santa Cruz, sc-516608), at the same concentration as the primary antibody. 

### 4.6. Immunohistochemistry for TLR-2 

Mid-modiolar cochlear cryosections were washed with 0.1 PBS (3 × 10 min), permeabilized with 1% Triton X-100 and blocked with 10% normal goat serum (NGS) in 0.1 M PBS for 1 h at room temperature. Mid-modiolar cochlear cryosections were incubated overnight at 4 °C with the rat anti-mouse TLR-2 monoclonal antibody (CD282; Invitrogen, Carlsbad, CA, USA, Product# 14-9021-82; 2.5 μg/mL) in 0.1% Triton X-100 and 10% NGS in 0.1M PBS. The following day, the sections were incubated in Alexa Fluor 488 goat anti-rat IgG (Invitrogen, Carlsbad, CA, USA, Product# A-11006; 4 μg/mL) for 2 h at room temperature. The cryosections were then washed three times with 0.1M PBS (2 × 10 min and the final 40-min wash), then mounted on microscope glass slides using FluoroshieldTM with DAPI mounting medium (Sigma-Aldrich).

### 4.7. Imaging 

The Olympus FV1000 confocal microscope was used to image the cochlear cryosections. The images were processed with FluoView software (version 2.0c, Olympus, Tokyo, Japan). A Z-stack was taken in all areas at 2 μm/slice to capture the full depth of the cryosection. Low-resolution images of Iba1+ cells were obtained using the 20× oil objective lens (UPLSAPO 20X O NA:0.85) and high-resolution images with 63× oil objective lens (UPLSAPO 100X O NA:1.40). Images were acquired at the pixel resolution of 0.618 μm/pixel for 20×, and 0.071 μm/pixel for 63×. The same microscope settings were applied to all images.

### 4.8. Semi-Quantitative Analysis of Immunofluorescence Intensity

Immunofluorescence intensity in the cochlea was assessed semi-quantitatively by measuring the pixel intensity in the regions of interest (ROI). The pixel intensity was measured using ImageJ (version1.46r) by converting RGB pixels to grey-scale/brightness values. ImageJ analysis was performed to quantify the intensity of immunofluorescent signals from a Z-stack of images. Images from the channel of interest were processed using the Gaussian Blur, and these blurred images were used as “background”, which was subtracted by the “image calculator” function of ImageJ to calibrate grey values across sections and animals. Then, the image stack was processed by thresholding into binary images. The threshold value for binary conversion was determined using the average of the mean and minimum error method in ImageJ. The mean grey values were calculated from four mid-modiolar cochlear cryosections per animal (5 mice per diet group). Immunofluorescence intensity in HFD mice was normalised to the average signal in the CD group for each ROI and presented as a fold change.

### 4.9. Gene Expression Studies

Quantitative RT-PCR studies were used to assess changes in mRNA expression levels of pro-inflammatory and anti-inflammatory mediators in all mice. We analysed gene expression levels of TLR2, TLR4, TNF-α, IL-6, ICAM1, and TGF-β using cochlear mRNA samples obtained from 12 mice (6 per diet group). 

The mRNA extraction was performed using the Dynabeads^®^ mRNA DIRECT™ Kit (Ambion^®^, Life Technologies, Oslo, Norway). Briefly, the mouse cochlea was homogenized in lysis buffer (100 mM Tris-HCl pH 7.5, 500 mM LiCl, 10 mM EDTA pH 8, 1% LiDS, 5mM dithiothreitol, RNase inhibitor) using an autoclaved mini-pestle. The tissue lysate was then transferred to a sterile tube containing pre-conditioned Dynabeads^®^ to allow the poly-A-tails of the mRNA to anneal to the oligo(dT)_25_ primers coupled to the Dynabeads^®^. The mRNA and Dynabeads^®^ were resuspended in ice-cold elution buffer (10 mM Tris HCl) and then heated at 67 °C for two minutes to separate mRNA. First-strand complementary DNA (cDNA) was synthesized through reverse transcription from the extracted mRNA using SuperScript^®^ IV First-Strand Synthesis System (Invitrogen, Carlsbad, CA, USA. 

The transcript levels of inflammatory mediators in the cochleae were quantified by real-time quantitative RT-PCR (qRT-PCR). qRT-PCR was performed in MicroAmp Optical 384-well reaction plates using TaqMan^®^ Universal PCR Master Mix (Invitrogen, Auckland, New Zealand), unlabelled PCR primers, and pre-designed FAM-labelled TaqMan MGB probes proprietary to Invitrogen (Carlsbad, CA, USA, TaqMan^®^ Gene Expression Assays). As a template, 1 μL of sample cDNA was added to a total reaction volume of 12.5 μL. The samples were tested in duplicate, and data were expressed as a mean of two duplicates. Negative controls without a template or reverse transcriptase were included in every PCR run. Quantitation of a house-keeping gene, mouse β-actin, was performed for all samples as an endogenous reference. Amplification and fluorescence detection were carried out using the QuantStudio 5 Quantitative Real-Time PCR system and analyzed using the QuantStudio 5 Design & Analysis Software (Applied Biosystems, Waltham, MA, USA). The thermal cycling protocol included 2 min at 50 °C, 10 min at 95 °C and 40 cycles of 15 s at 95 °C and 1 min at 60 °C. Gene expression levels of inflammatory mediators were normalised to the reference β-actin gene expression. Quantitative analysis of gene expression in mice on an HFD was performed using the 2(^-ΔΔCt^) method (45) and expressed as fold change relative to CD mice. 

### 4.10. Data Analysis

Statistical analysis was performed using the Prism Graph Pad software (version 9.02). All data were tested for normality using the Shapiro–Wilks test. An unpaired Student’s *t*-test was used to examine the statistical difference between HFD and CD groups, and a *p*-value < 0.05 was considered statistically significant. Data were presented as mean ± standard error of the mean (SEM).

## 5. Conclusions

This study provides preliminary evidence that a chronic high-fat diet induces inflammatory changes in the mouse cochlea. These changes include upregulation of ICAM1, IL-6Rα, and TLR2 at the protein level and the recruitment of Iba1+ macrophages in the cochlea. However, no significant changes of pro-inflammatory and anti-inflammatory biomarkers were observed at the gene expression level. This could suggest that the cochlea is in a transitory phase from the pro-inflammation to the resolution phase, which aims to protect the cochlea from the detrimental effects of chronic inflammation. The evidence gathered from this study provides a fertile ground further to investigate these inflammatory changes in the inner ear and establish the impact of an HFD on cochlear health and disease. Further studies are required to establish the effect of HFD-induced inflammation on auditory function and cochlear tissue damage using different animal models and the duration of HFD. It is also necessary to determine dynamic changes in gut microbiota and link these changes to the inflammatory status of the cochlea and the rest of the body. 

## Figures and Tables

**Table 1:** Bodyweight measurements in CD-1 mice.

	Control Diet	High Fat Diet	*p*-Value (*t*-Test)
**Baseline BW (g)**	32.6 ± 1.1	33.1 ± 1.5	0.7808
**Post-diet BW (g)**	35.4 ± 1.9	42.7 ± 1.4	0.0051

**Table 2:** Primary antibody information for conjugated antibodies.

Conjugated Antibody	Company	Product#	Concentration	Dilution	Specificity
IL-6Rα (D-8) Alexa 488	Santa Cruz Biotechnologies	Sc-374259	200 μg/mL	1:100 (2 μg/mL)	mouse
ICAM1 (6.5B5) Alexa 488	Santa Cruz Biotechnologies	Sc-18853	200 μg/mL	1:100 (2 μg/mL)	mouse
Iba1 (1022-5) Alexa 594	Santa Cruz Biotechnologies	Sc-32725	200 μg/mL	1:100 (2 μg/mL)	mouse

## Data Availability

Not applicable.

## Supplementary Materials

The following supporting information can be downloaded at: https://www.mdpi.com/article/10.3390/ijms23095179/s1.
