# Supplementary material for: A High-Fat Diet Induces Low-Grade Cochlear Inflammation in CD-1 Mice
Source: Int J Mol Sci. 2022 May 6;23(9):5179. doi: 10.3390/ijms23095179 (PMC9101486; doi:10.3390/ijms23095179)
Supplement: Supplementary file 1 [file ijms-23-05179-s001.zip › ijms-1694066-supplementary/Supplementary Figure S1.pdf]

Supplementary Figure S1. Formulation and caloric information of a high-fat diet (Research Diets, New Brunswick, NJ, D12492I)

Formulation

Class description	Ingredients	Grams
Protein	Casein, Lactic, 30 Mesh	200.00 g
Protein	Cystine, L	3.00 g
Carbohydrate	Lodex 10	125.00 g
Carbohydrate	Sucrose, Fine Granulated	72.80 g
Fiber	Solka Floc, FCC200	50.00 g
Fat	Lard	245.00 g
Fat	Soybean Oil, USP	25.00 g
Mineral	S10026B	50.00 g
Vitamin	Choline Bitartrate	2.00 g
Vitamin	V10001C	1.00 g
Dye	Dye, Blue FD&C #1, Alum. Lake 35-42%	0.05 g
	Total:	773.85 g

Caloric Information Physiological Fuel Values

Protein:	**20** % Kcal
Fat:	**60** % Kcal
Carbohydrate:	**20** % Kcal
Energy density:	**5.21** Kcal/g
