# Supplementary material for: A High-Fat Diet Induces Low-Grade Cochlear Inflammation in CD-1 Mice
Source: Int J Mol Sci. 2022 May 6;23(9):5179. doi: 10.3390/ijms23095179 (PMC9101486; doi:10.3390/ijms23095179)
Supplement: Supplementary file 1 [file ijms-23-05179-s001.zip › ijms-1694066-supplementary/Supplementary Figure S2.pdf]

Supplementary Figure S2. Formulation and caloric information of control diet (Research Diets, New Brunswick, NJ, D12450J)

Class description	Ingredients		Grams
Protein	Casein, Lactic, 30 Mesh		200.00 g
Protein	Cystine, L		3.00 g
Carbohydrate	Starch, Corn		506.20 g
Carbohydrate	Lodex 10		125.00 g
Carbohydrate	Sucrose, Fine Granulated		72.80 g
Fiber	Solka Floc, FCC200		50.00 g
Fat	Soybean Oil, USP		25.00 g
Fat	Lard		20.00 g
Mineral	S10026B		50.00 g
Vitamin	Choline Bitartrate		2.00 g
Vitamin	V10001C		1.00 g
Dye	Dye, Yellow FD&C #5, Alum. Lake 35-42%		0.04 g
Dye	Dye, Blue FD&C #1, Alum. Lake 35-42%		0.01 g
		Total:	1055.05 g

Caloric Information Physiological Fuel Values

Protein:	**20** % Kcal
Fat:	**10** % Kcal
Carbohydrate:	**70** % Kcal
Energy density:	**3.82** Kcal/g
